# Three-in-One Simultaneous Extraction of Proteins, Metabolites and Lipids for Multi-Omics

**DOI:** 10.3389/fgene.2021.635971

**Published:** 2021-04-15

**Authors:** Jianing Kang, Lisa David, Yangyang Li, Jing Cang, Sixue Chen

**Affiliations:** ^1^College of Life Science, Northeast Agricultural University, Harbin, China; ^2^Department of Biology, University of Florida, Gainesville, FL, United States; ^3^University of Florida Genetics Institute, Gainesville, FL, United States; ^4^College of Horticulture, Shenyang Agricultural University, Shenyang, China; ^5^Plant Molecular and Cellular Biology Program, University of Florida, Gainesville, FL, United States; ^6^Proteomics and Mass Spectrometry, Interdisciplinary Center for Biotechnology Research, University of Florida, Gainesville, FL, United States

**Keywords:** multi-omics, 3-in-1 method, proteomics, metabolomics, lipidomics, *Arabidopsis*, disease

## Abstract

Elucidation of complex molecular networks requires integrative analysis of molecular features and changes at different levels of information flow and regulation. Accordingly, high throughput functional genomics tools such as transcriptomics, proteomics, metabolomics, and lipidomics have emerged to provide system-wide investigations. Unfortunately, analysis of different types of biomolecules requires specific sample extraction procedures in combination with specific analytical instrumentation. The most efficient extraction protocols often only cover a restricted type of biomolecules due to their different physicochemical properties. Therefore, several sets/aliquots of samples are needed for extracting different molecules. Here we adapted a biphasic fractionation method to extract proteins, metabolites, and lipids from the same sample (3-in-1) for liquid chromatography-tandem mass spectrometry (LC-MS/MS) multi-omics. To demonstrate utility of the improved method, we used bacteria-primed *Arabidopsis* leaves to generate multi-omics datasets from the same sample. In total, we were able to analyze 1849 proteins, 1967 metabolites, and 424 lipid species in single samples. The molecules cover a wide range of biological and molecular processes, and allow quantitative analyses of different molecules and pathways. Our results have shown the clear advantages of the multi-omics method, including sample conservation, high reproducibility, and tight correlation between different types of biomolecules.

## Introduction

Systems biology, the comprehensive study of biological components and their interactions within a cell or a tissue, is indispensable toward understanding complex cellular functions and processes. Multi-omic measurements and integration of the resulting information can transform our understanding of complex biological systems (Dai and Chen, 2012; He et al., 2012; Mostafa et al., 2016; Meng et al., 2019). Multiple layers of information (DNA, RNA, protein, metabolite, and lipid) can provide important insights into cellular molecular networks. In recent years, rapid progress has been made in genomics and transcriptomics. Nevertheless, proteomics, metabolomics, and lipidomics have emerged as cornerstones in the field of systems biology because the essential information at protein, metabolite, and lipid levels cannot be predicted or deduced from genomics and transcriptomics (He et al., 2012; Geng et al., 2016, 2017; Mostafa et al., 2016; Meng et al., 2019).

**FIGURE 1:**
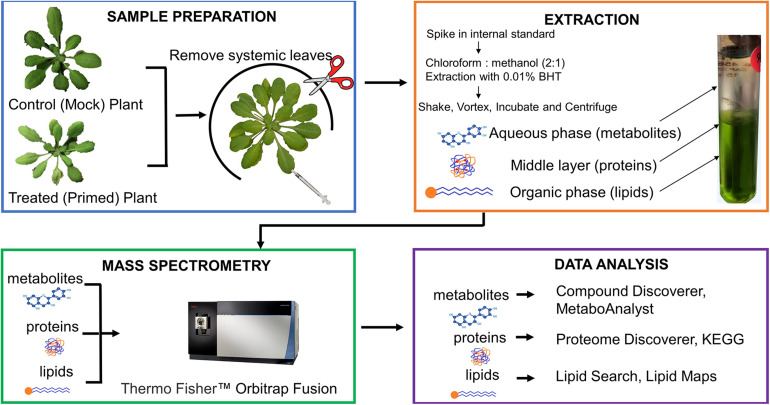
Diagram of 3-in-1 sample preparation method for profiling proteins, metabolites, and lipids from control and primed *Arabidopsis* leaves. The biphasic fractionation separates three types of biomolecules simultaneously, which are analyzed on the same mass spectrometry platform. The data are also analyzed using the same vendor’s software. A more detailed workflow of the extraction is shown in Figure 2.

**FIGURE 2:**
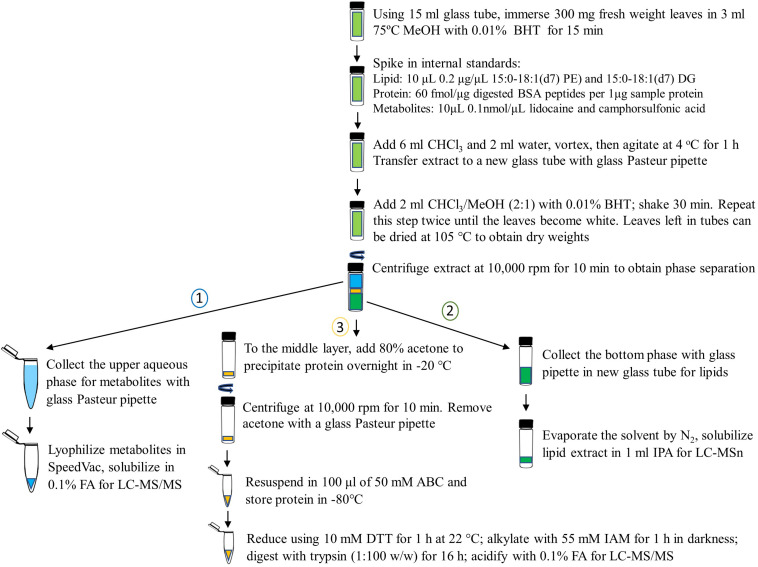
Detailed workflow of 3-in-1 sample extraction of proteins, metabolites, and lipids from control and primed *Arabidopsis* leaves. A chloroform/methanol/water extraction is used to separate the three fractions and each layer is carefully isolated using supplies of glass materials to avoid plastic contaminants in samples. The order of fractionated is important and labeled. Butylated hydroxytoluene (BHT) is added at the start of the extraction to avoid oxidation of lipids during the procedure. MeOH, methanol; PE, phosphatidylethanolamine; DG, diacylglycerol; BSA, bovine serum albumin; CHCl_3_, chloroform; FA, formic acid; LC-MS/MS, liquid chromatography tandem mass spectrometry; IPA, isopropanol; ABC, ammonium bicarbonate; DTT, dithiothreitol; IAM, iodoacetamide.

To conduct multi-omics, aliquots of the same sample are required for different extraction procedures optimized for different biomolecules. In addition to increased effort inherent to different parallel sample handling, the required sample amounts for multiple extractions are often not available. Meanwhile, the multi-components extracted from parallel sets of replicates can decrease consistency and comparability when performing multi-omics integration. Consequently, a versatile extraction method providing robust and reliable recovery of the molecular components from a single sample is desirable. Such a method decreases sample handling time and thus increases throughput. Importantly, it conserves critical samples and improves data accuracy and comparability because different molecules are all derived from the same sample. Common methods employed for fractionated extractions are based on a two-phase lipid extraction method developed in 1957 (Folch et al., 1957). It uses chloroform/methanol/water partitioning of polar and hydrophobic metabolites and was designed to increase the purity of lipids. Here we modified and optimized this method to obtain high quality proteins, metabolites, and lipids from a single sample, as a 3-in-1 method (Figures 1, 2). This method can be easily applied to many types of materials. It should be noted that when applying to other sample types, the amount of samples may vary based on the types of samples and their water content, etc. Regardless of the source material, proteins, lipids, and metabolites have the same physicochemical properties, therefore, this method has broad application potential.

To test the utility of our 3-in-1 extraction method, we used leaves of *Arabidopsis thaliana* (WS ecotype) that had been primed by a pathogenic *Pseudomonas syringae pv. tomato* DC3000 (*Pst* DC3000). Systemic Acquired Resistance (SAR), a salicylic acid (SA)-dependent immune response, improves immunity of systemic tissues after prior localized exposure to a pathogen. *Arabidopsis* knockout mutants defective in SAR response differ in disease resistance when compared to wild type plants. Here we used *Arabidopsis* wild type and a knockout mutant of a lipid transfer protein DIR1 (defective in induced resistance 1) to examine SAR in whole leaves. We have successfully used the 3-in-1 method and annotated 424 lipids, which cover most of the lipid classes. In addition, we have identified 1967 metabolites using a LC-MSn method, and obtained 1849 protein identifications. These results demonstrate the superior 3-in-1 method can greatly facilitate multi-omics studies in systems biology.

## Materials and Methods

### Plant Growth and Bacterial Injection

*Arabidopsis thaliana* wild type (WS ecotype) and *dir1* mutant (in WS background) were grown in 8 h light/16 h dark with a light intensity of 140 μmol/m^2^ s. One mature rosette leaf of the 5-week-old plants was injected with either *Pst* DC3000 in 10 mM MgCl_2_ (OD600 = 0.02) for treated plants or 10 mM MgCl_2_ for mock plants using a needleless syringe. Fully expanded distal rosette leaves that were not injected were collected at 48 h after infiltration and directly frozen in liquid nitrogen and stored in −80°C for 3-in-1 extraction. Three biological replicates of treated and three biological replicates of mock leaves were used.

### Multi-Omics Sample Preparation

Three hundred milligrams fresh weight leaves were quickly immersed in glass tubes with 3 ml pre-heated 75°C methanol (MeOH) and 0.01% butylated hydroxytoluene (BHT) and incubated for 15 min. Internal standards were added to each sample as follows: for proteins: 60 fmol bovine serum albumin (BSA) tryptic peptides per 1 μg sample protein; for metabolites: 10 μL 0.1 nmol/μL lidocaine and camphorsulfonic acid; and for lipids: 10 μL 0.2 μg/μL deuterium labeled 15:0-18:1(d7) phosphatidylethanolamine (PE) and 15:0-18:1(d7) diacylglycerol (DG).

For extraction of proteins, metabolites and lipids, 6 ml of chloroform and 2 ml of water (3:1, vol/vol) were added to each tube and 500 μl of MeOH was added to replenish the methanol that evaporated from boiling (Folch et al., 1957). Samples were vortexed at 4°C for 1 h. The liquid was transferred from the extracts to glass centrifuge tubes for further phase separation. To improve collection of all 3 components, 2 ml of chloroform/methanol (2:1 vol/vol) with 0.01% BHT was added to the leaves in the glass tubes and agitated for another 30 min at 4°C. This liquid was combined with the previous into the glass centrifuge tubes. This last extraction procedure was repeated twice on all the samples until the leaves appeared white. After extraction, leaves were dried at 105°C overnight and weighed for dry weights.

For phase separation, extracts were centrifuged at 10,000 rpm for 10 min at 4°C. First the upper (metabolites in MeOH) phase was collected and transferred to plastic 2 ml centrifuge tubes, then the bottom (lipids in chloroform) phase was removed and transferred to glass tubes, leaving the middle (protein) layer for protein collection. The lipid extract was evaporated by the Nitrogen gas and dried sample tube filled with nitrogen gas was placed at −80°C. The lipid extract was dissolved in 1 ml isopropanol (IPA) for LC-MS analysis. Metabolites were lyophilized to dryness, then the tubes were filled with argon and placed at −80°C. Metabolites were solubilized in 100 μl of 0.1% formic acid (FA) for LC MS/MS analysis.

### Protein Precipitation and Trypsin Digestion

Proteins were precipitated by addition of 80% acetone in the glass centrifuge tubes in −20°C. After 16 h, the samples were centrifuged at 10,000 rpm for 10 min at 4°C. After removing acetone, proteins were resuspended in 100 μl of 50 mM ABC, reduced using 10 mM dithiothreitol (DTT) for 1 h at 22°C, and then alkylated with 55 mM iodoacetamide (IAM) for 1 h in darkness. The samples were digested with trypsin (1:100 w/w) for 16 h. All the samples were acidified by addition of 0.1% FA to stop the digestion and stored at −80°C.

### Liquid Chromatography Mass Spectrometry (LC-MS) and Omics Data Analysis

Untargeted metabolomic, lipidomic, and proteomic methods were run on an Orbitrap Fusion Tribrid mass spectrometer (Thermo Fisher Scientific, Bremen, Germany). A Vanquish^TM^ UHPLC was used for lipids and metabolites, and an Easy-nLC was used for peptides. An Accucore C18 (100 mm × 2.1 mm, 2 μm) column and an Acclaim C30 (2.1 mm × 250 mm, 3 μm) were used for metabolites and lipids, respectively. The column chamber temperature was 55°C, and the pump flow rate was 0.45 ml/min. For metabolomics, solvent A (0.1% FA) and solvent B (0.1% FA and 99.9% acetonitrile) were used. The LC gradient is set to 0 min: 1% of solvent B, 5 min: 1% of B, 6 min: 40% of B, 7.5 min: 98% of B, 8.5 min: 98% of B, 9 min: 0.1% of B, 10 min stop run. To enhance identification, an AcquireX MSn data acquisition strategy was used which employs replicate injections for exhaustive sample interrogation and increases the number of compounds in the sample with distinguishable fragmentation spectra for identification (David et al., 2021). Pooled samples were created using equal volumes of all the samples for quality control, and were run after each sample set. Electrospray ionization spray voltage for positive ions was 3500 and for negative ions was 2500. Sheath gas was set to 50, auxiliary gas was set at 1 and sweep gas was set to 1. The ion transfer tube temperature was set at 325°C and the vaporizer temperature was set at 350°C. Full MS1 used the Orbitrap mass analyzer with a resolution of 120,000, scan range (m/z) of 55–550, maximum injection time (MIT) of 50, automatic gain control (AGC) target of 2e5, 1 microscan, and RF lens set to 50. For lipidomics, solution A consisted of 0.1% FA, 10 mM ammonium formate, and 60% acetonitrile. Solution B consisted of 0.1% FA, 10 mM ammonium formate, and 90:10 acetonitrile: isopropyl alcohol. The LC gradient is set to 0 min: 32% of solvent B (i.e., 68% of solvent A), 1.5 min: 45% of B, 5 min: 52% of B, 8 min: 58% of B, 11 min: 66% of B, 14 min: 70% of B, 18 min: 75% of B, 21 min: 97% of B, 26 min: 32% of B, 32 min stop run. Full MS1 used the Orbitrap ion trap mass analyzer with a resolution of 70,000, 1 microscan, AGC target set to 1e6, and a scan range from 200 to 2000 m/z for positive and negative polarity. The dd-MS^2^ scan used 1 microscan, resolution of 35,000, AGC target 5e5, MIT of 46 ms, and loop count of 3.

The column used for peptides was the Acclaim PepMap^TM^ 100 pre-column (75 μm × 2 cm, nanoViper C18, 3 μm, 100 A) combined with an Acclaim PepMap^TM^ RSLC (75 μm × 25 cm, nanoViper C18, 2 μm, 100 A) analytical column. The LC runs a linear gradient of solvent B (0.1% FA, 99.9% Acetonitrile) from 1 to 30% for 90 min at 250 nL/min. The solvent A was 0.1% FA. The MS was operated in data-dependent acquisition mode with a cycle time of 3 s. Eluted peptides were detected in the Orbitrap MS at a 120,000 resolution with a scan range of 350–1800 m/z. Most abundant ions bearing 2–7 charges were selected for MS/MS analysis. AGC for the full MS scan was set as 2e5 with MIT as 50 ms, and AGC Target of 1e4 and MIT of 35 ms were set for the MS/MS scan. The normalized collision energy was 35, and ions were detected with an Ion Trap detector. A dynamic exclusion time of 30 s was applied to prevent repeated sequencing of the most abundant peptides.

Proteome Discoverer^TM^ 2.4, Compound Discover^TM^ 3.0, and Lipid Search 4.1^TM^ software (Thermo Fisher Scientific, Bremen, Germany) were used for proteomics, metabolomics and lipidomics data analyses, respectively (Figure 1). Software scoring parameters used for metabolite, lipid, and protein identifications are briefly described here with references provided to previous publications with more details (Geng et al., 2016, 2017; Breitkopf et al., 2017). Briefly, for proteomic data analysis, MS/MS spectra were searched against *Arabidopsis* TAIR10 database with 10 ppm mass tolerance for MS1 and 0.02 Da tolerance for MS2, two missed cleavage sites, fixed modification of cysteine carbamidomethylation (+57.021), and dynamic modifications of methionine oxidation (+15.996). Peptide confidence level was set at 1% false discovery rate with at least two unique peptides. Relative protein abundance in treated and mock samples was measured using label-free quantification in the Proteome Discoverer^TM^ 2.4. For metabolomics data, metabolite identification included predicting compositions, searching mzCloud spectra database, and assigning compound annotations by searching ChemSpider, Pathway mapping to KEGG and Metabolika pathways was used for functional analysis. The metabolites were scored by applying mzLogic and the best score was kept. Peak areas were normalized by the positive and negative mode internal standards (lidocaine and camphorsulfonic acid, respectively) (Geng et al., 2017). For lipidomics data, raw files from three replicates of mock and treated were uploaded to Lipid Search 4.1^TM^ for annotation of lipids found in all the samples. A mass list was generated for uploading to Compound Discover^TM^ 3.0 Software. This mass list was used for metabolite identification along with predicted compositions, mzCloud database matching, and compound annotations. Lipid Search scoring algorithms considering lipid fragmentation ions related to headgroup, fatty acids and backbone, as well as precursor and product ion accuracy of 5 ppm were used. Peak areas were normalized by median-based normalization.

Statistical analyses were done by normalizing peak areas by internal standards spiked in the samples. The average areas of three biological replicates of each group were compared as a ratio and two criteria were used to determine significantly altered components: (1) *p*-value from an unpaired student’s *t*-test less than 0.05, and (2) increase or decrease of 2-fold (*dir1* primed/wild type primed) (Supplementary Table 1). All protein MS raw data and search results have been deposited to the ProteomeXchange Consortium via the PRIDE partner repository with the data set PXD023094. All the metabolomics and lipidomics MS raw data and search results have been deposited to the MetaboLights repository with the data set identifier MTBLS2303.

## Results and Discussion

The multi-omics sample preparation workflow that we have developed has allowed us to increase the number of lipids, proteins, and metabolites identified from a single sample (Figure 1). Previous extraction methods applied to *Arabidopsis* leaves identified 1987 proteins (Nakayasu et al., 2016), 2638 proteins (Salem et al., 2016), 150 metabolites and 200 lipid species (Salem et al., 2016, 2017; Table 1). Our method was able to identify 1849 confident proteins with 2 or more unique peptides at FDR of less than 1%. Our method greatly increases the number of polar metabolites to 1967, and non-polar lipids to 424 lipid species (Table 1). This represents a more than 10-fold increase in the identified metabolites and more than twice the number of identified lipid species, when compared to previous *Arabidopsis* papers (Salem et al., 2016, 2017). The number of identified proteins in this work appears to be lower than reported in a previous paper (Salem et al., 2016), but we used stringent criteria for high confidence. Otherwise, we could have identified 2778 proteins (Table 1). We also compared our method to other three-part extraction methods developed for mammalian cell lines (Coman et al., 2016; Nakayasu et al., 2016). Again, our method stands out considering the large numbers of identified polar and non-polar metabolites. While the overall number of identified proteins in our samples is lower than those reported in Coman et al. (2016) and Nakayasu et al. (2016) (Table 1), we are fully aware that such a comparison may not be sensible because of species and protein database differences. For instance, the mouse proteome is larger with 55,152 entries in UniProt, while the TAIR10 database contains 35,386 entries (Zhang et al., 2019). Nevertheless, we can reasonably expect that our 3-in-1 method will lead to valuable results when applied to mammalian cells. Among all the 3-in-1 methods in Table 1, our method is most similar to Nakayasu et al. (2016), which used human epithelial Calu-3 cells. For *Arabidopsis*, they only reported identification of 1987 proteins using an-house software. Since it is not clear about their FDR and unique peptide criteria, it may be reasonable to assume that our protein data of 2778 proteins (with 1% FDR) and 1894 proteins (after applying additional two unique peptide filter) are comparable, if not better. Importantly, we identified nearly 20 times more metabolites and more than twice the lipid species (Table 1 and Supplementary Table 2). Here are some technical improvements in our method: (1) we added a reductant at the first step to preserve lipids and extracted for longer time; (2) we did three chloroform/methanol extraction steps until the leaves looked white in color, while Nakayasu et al. (2016) only extracted one time; (3) we lyophilized the fractions of metabolites and lipids (under nitrogen gas) before reconstitution and LC-MSn, while they collected the lipid and metabolite layers directly into autosampler vials; (4) we used a new AcquireX LC-MSn data acquisition strategy (David et al., 2021), which enhanced the coverage of metabolome and lipidome; (5) their metabolomics was done using GC-MS, which is known to cover a small number of central metabolites (Gowda and Djukovic, 2014; Geng et al., 2017); and (6) this work may have also benefited from the use of Compound Discoverer software with access to a large MzCloud database of MS2 spectra.

**TABLE 1:** Comparison of the three-in-one method in this study with previously published targeted and three-in-one methods.

	Proteins		Metabolites		Lipids		Simultaneous extraction of proteins, metabolites, and lipids				
References	Zybailov et al., 2009	Niehl et al., 2013	Fiehn et al., 2000	Wu et al., 2018	Higashi et al., 2015	Kehelpannala et al., 2020	Coman et al., 2016^a^	Nakayasu et al., 2016^a^	Salem et al., 2016	Salem et al., 2017	This work
Materials	*Arabidopsis* ecotype Col-0	*Arabidopsis* ecotype Col-0	Arabidopsis ecotypes Col-2 and C24	*Arabidopsis* ecotype Col-0	*Arabidopsis* ecotypes Col-0 and Nossen	*Arabidopsis* ecotype Col-0	Mouse bone marrow cells	*Arabidopsis* Human epithelial Calu-3 cells	*Arabidopsis* ecotype Col-0	*Arabidopsis* ecotype Col-0	*Arabidopsis* ecotype WS
Extraction	Tris buffer with 5% SDS	Trizol and acetone precipitation	Chloroform: methanol: H_2_O	Methyl-tert-butyl-ether: methanol: H_2_O	Chloroform: methanol: H_2_O	Chloroform: methanol: H_2_O	Methyl-tert-butyl- ether: methanol: H_2_O	Chloroform: methanol: H_2_O	Methyl-tert-butyl- ether: methanol: H_2_O	Methyl-tert-butyl- ether: methanol: H_2_O	Chloroform: methanol: H_2_O
Fractional on	Gel electrophoresis into 12 fractions	Gel electrophoresis into 8 fractions	Lipophilic and polar phases	Aqueous phase	Lipophilic phase	Lipophilic phase	SIMPLEx containing 3 phases	MPLEx containing 3 phases	Polar and non-polar liquid and liquid fractional	Polar and non-polar liquid and liquid fractional on	Triphasic fractionation
Chromato- graphy	Ultimate LC with 90 min gradient	Picotip with 50 min LC gradient	Gas chromatography 8000	Waters Acquity LC with 44 min gradient	Shimadzu LC with 40 min gradient	Agilent 1290 LC with 30 min gradient	Ultimate 3000 LC with 45 min gradient	Nano-/Cap-LC with 90 min gradient, Agilent GC-MS	Ultimate LC with 110 min gradient	Ultimate LC with 110 min gradient	Ultimate LC with 90 min gradient
Mass spectrometer	LTQ Orbitrap MS/MS	LTQ Qrbitrap MS/MS	Voyager mass spectrometer	Exactive Qrbitrap MS	Ion trap-Time-of-Flight (TOF) MS	Quadrupole-TOF MS/MS	LTQ Orbitrap Velos and QTRAP 6500 MS/MS	LTQ-Orbitrap Velos MS/MS	Q-Exactive Orbitrap MS/MS	Q-Exactive Orbitrap MS/MS	Orbitrap Fusion Tribrid MSn and AquireX
Software	Mascot 2.2	Mascot 2.3	MassLab FindTarget and Pirouette	REFINER MS 10.0	Profiling Solution and in-house Perl script	MS-DIAL	Progenesis 4.1	VIPER (in-house)	Mascot 2.5	Mascot 2.5	Proteome Discoverer 2.4
							MultiQuant 3.0	Metabolite Detector	Target Search	Target Search	Compound Discover 3.0
							Chipsoft 8.3.1	LIQUID (in-house)	Progenesis QI2.2	Progenesis QI 2.2	Lipid Search 4.1
Level^b^	2	2	1	2	1 and 2	2	1 and 2	2	2	2	1 and 2
Identification	2800 proteins	1474 proteins	326 metabolites	123 metabolites	66 lipids	208 lipids	3327 proteins	1987/2670 proteins^c^	2638 proteins	Not available	2778/1849 proteins^d^
							75 metabolites	51 metabolites	150 metabolites	50 metabolites	1967 metabolites
							360 lipids	236/171 lipids^e^	200 lipids	200 lipids	424 lipids

*Our three-part extraction method is compared to other single component extraction methods and to other three-component extraction methods. Extraction technique, sample preservation, instrumentation method, and data analysis software all play a role in the improved profiling of proteins, metabolites, and lipids. a. this paper used mammalian cells. All other samples are Arabidopsis leaves. b. Level 1, Authentic standards (identification); Level 2, MS/MS data matching to library/database. c. 1987 proteins from Arabidopsis (of unknown ecotype), 2670 proteins identified from Human epithelial Calu-3 cells. d. 2778 proteins identified only with 1% FDR, and 1894 proteins after applying a two unique peptide filter. e. while it is written in the paper text that there were 236 lipids, only a total of 171 lipids could be found in the Supplementary Tables.*

Here we also compared our 3-in-1 extraction method to previously published methods targeted to a single component, including proteins (Zybailov et al., 2009; Niehl et al., 2013), metabolites (Fiehn et al., 2000; Wu et al., 2018), and lipids (Higashi et al., 2015; Kehelpannala et al., 2020). We found that our method allows for similar numbers of identified proteins, and increased numbers of metabolites and lipids when compared to these single component extraction methods (Table 1). Please note, that protein work mentioned in Table 1 include gel-based sample prefractionation step to improve coverage, but in our study we obtained similar numbers of proteins without this fractionation step. We can attribute the improved extraction and identification of metabolites, lipids, and proteins to three factors: 1. advanced instrumentation by using the Orbitrap tribrid mass spectrometer; 2. deep sampling and fragmentation of analytes using the AcquireX technology resulting in improved level 2 identification by MS2 and MS3; and 3. preservation of each layer by using Nitrogen gas for evaporation of chloroform in lipid samples and addition of reductant to avoid lipid and metabolite oxidation, as well as avoiding disruption of middle layer to preserve for protein precipitation in acetone and the use of only glass materials to avoid plastic contaminations during extraction (Table 1 and Figure 2). This procedure also requires careful removal of each component layer so as not to disrupt and disperse the middle layer that contains the proteins. This was achieved by avoiding agitation of the glass tube after removing from the centrifuge and by carefully sliding the glass pipette along the side of the tube to draw off the metabolite and lipid layers sequentially, leaving the protein layer intact (Figure 2).

**FIGURE 3:**
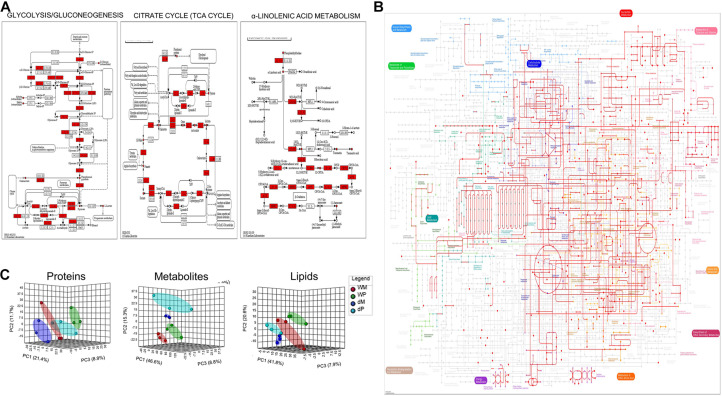
Evaluation of KEGG pathway coverage, data quality, and performance improvement with the 3-in-1 extraction method. **(A)** Enhanced coverage of specific molecular pathways by the identified proteins, metabolites, and lipids. In The red-colored boxes represent identified proteins and the red-colored circles are lipids and metabolites. **(B)** Mapping of the quantified proteins, metabolites, and lipids onto the KEGG metabolic pathways. **(C)** Principal component analysis (PCA) of relative levels of proteins, metabolites, and lipids obtained from three biological replicates under the four experimental conditions (WM, wild type mock; WP, wild type primed; dM, *dir1* mock; dP, *dir1* primed).

Increased identification of proteins, metabolites and lipids is essential for understanding the interconnected molecular networks that mediate cellular responses. Figure 3A shows that different molecules (proteins, metabolites, and lipids) from a specific pathway can be examined together to gauge potential regulations and activities of the pathway. This is important because protein abundance data do not reflect the activity of the protein, but when combined with the information for metabolites and lipids, the activities of enzymes leading to synthesis of the metabolites can be deduced. Figure 3B shows that the identified proteins from *Arabidopsis* leaves cover a wide range of molecular pathways (129 out of 541 KEGG pathways), in addition to pathways covered by the identified metabolites and lipids, highlighting the complementary nature of different “omics.” In Figure 3C, principal component analysis shows unsupervised clustering of wild type samples and mutant samples separately, and also that mock versus treated samples grouping together for proteins, metabolites and lipids. The results clearly indicate high reproducibility of the 3-in-1 method and its application to capturing biological differences related to *Arabidopsis* systemic acquired resistance.

When comparing to previous methods (Table 1), our new method clearly stands out in the high coverage of metabolome and lipidome. For example, both low abundant (methionine, tryptophan, and tyrosine) and high abundant amino acids (arginine and glutamic acid) in plants (Kumar et al., 2017) were identified. In addition, metabolites with a variety of chemical properties were covered, including polar (e.g., glutamine and tyrosine), non-polar (e.g., methionine), aromatic amino acids (e.g., tryptophan and tyrosine), cofactors (e.g., NAD^+^, ATP), and plant hormones (e.g., SA and jasmonic acid). Moreover, lipids also spanned a range of lipid classes and different concentrations in the cells. They include major lipid classes, such as glycerolipids: monoradylglycerolipids (MG), diradylglycerolipids (DG), and triradylglycerolipids (TG); glycerophospholipids: glycerophosphoserines (PS), glycerophosphoinositols (PI), glycerophosphoglycerols (PG), glycerophosphoethanolamines (PE), glycerophosphocholines (PC), lyso-glycerophosphoethanolamines (LPE); sphingolipids: ceramides (Cer); and galactolipids: monogalactosyldiacylglycerol (MGDG), digalactosyldiacylglycerol (DGDG), and digalactosylmonoacylglycerol (DGMG). Interestingly, the relative abundances of the lipid classes correlate well with those detected in previous publications (Supplementary Table 2), in spite the ecotype differences between this study and the other studies (Table 1).

**FIGURE 4:**
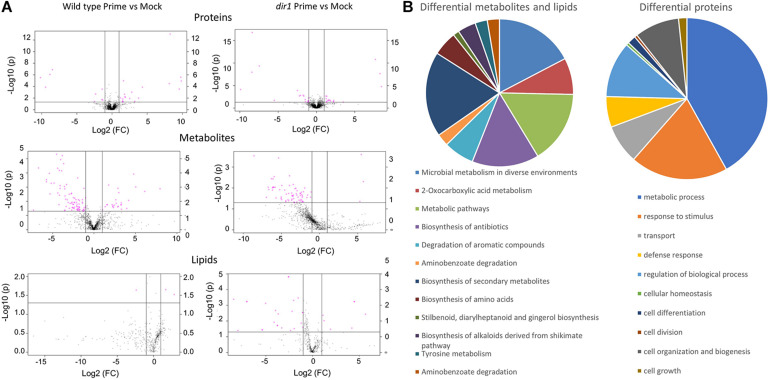
Significant changes of proteins, metabolites, and lipids in *Arabidopsis* leaves of wild type and *dir1* mutant primed by *Pst DC 3000* treatment. **(A)** Volcano plots displaying differential changes of proteins, metabolites, and lipids in wild type and *dir1* mutant. Pink dots indicate differential molecules. **(B)** Biological functions of the differential metabolites/lipids and proteins in wild type versus *dir1* primed leaves.

A successful multi-omics study should not only allow for large-scale discovery of biomolecules at different abundances, but also uncover meaningful biological processes and significance. Here we employ the 3-in-1 method in a proof-of-concept study to measure changes of proteins, metabolites and lipids from each sample during SAR. A volcano plot of the protein, metabolite, and lipid data from wild type SAR (primed/control) versus SAR in the *dirl* mutant showed many differential molecular changes with significant *p*-values of less than 0.05 (Figure 4A). The method also showed decent reproducibility even with biological replicate samples. Of the 113 differentially abundant proteins between *dir1* primed/WS primed, 112 had coefficient of variation (CV) less than 20%. Of the 135 differential metabolites and 15 lipids, they were 91 and all 15 less than 20%, respectively. Differential metabolites and lipids were grouped and mapped to KEGG pathways and differential proteins were separately mapped to KEGG pathways (Figure 4B). Interestingly, the largest group of differential proteins mapped to metabolic process and metabolic pathways was the second most abundant biological process for the differential metabolites (Figure 4B). Protein differences in the *dir1* primed versus wild type primed plants indicate that the altered *dir1* defense responses may account for its susceptibility when compared to wild type plants. Proteins in response to stimulus and defense response pathways were the second and sixth most abundant groups, respectively (Figure 4B). When examining metabolites and lipids that were different between the *dir1* and wild type primed leaves, we found the largest groups related to biosynthesis of secondary metabolites, biosynthesis of antibiotics, and biosynthesis of amino acids as the first, fourth, and seventh most abundant groups, respectively. Secondary metabolites and amino acids play well-known roles in plant defense responses (Zeier, 2013; Rojas et al., 2014; Kadotani et al., 2016; Erb and Kliebenstein, 2020). Additionally, biosynthesis of antibiotics can be correlated to defense response against the biological pathogen *Pst* during priming.

**FIGURE 5:**
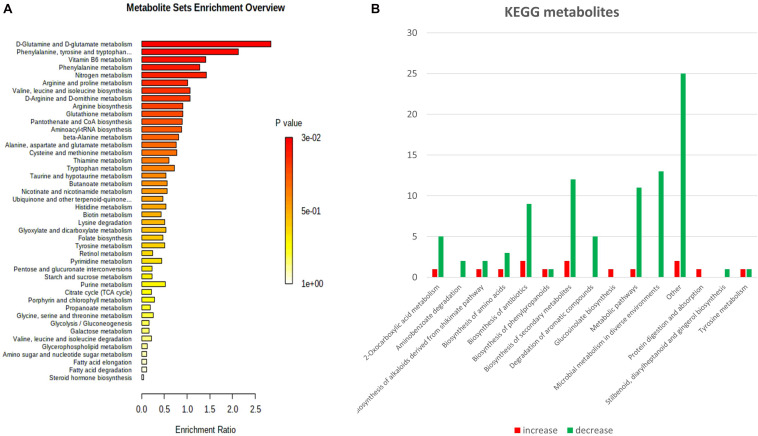
Enriched pathways and differential metabolites and lipids in *Arabidopsis* leaves of wild type and *dir1* mutant primed by *Pst* DC 3000 treatment. **(A)** MetaboAnalyst analysis of pathway enrichment for metabolites and lipids. **(B)** KEGG pathways of metabolites/lipids that are differentially abundant in wild type versus *dir1* primed leaves.

To further investigate the roles of the differential metabolites and lipids, we performed a pathway enrichment analysis (Figure 5A), revealing enrichment of multiple amino acid metabolic pathways including: glutamine and glutamate, phenylalanine, tyrosine, tryptophan, arginine, proline, valine, leucine, isoleucine, and lysine metabolism (Figure 5A). They were largely decreased in the susceptible *dir1* mutant in the category of amino acid biosynthesis (Figure 5B). Interestingly, the protein level changes corroborate the metabolomics data (Figure 5A), indicating translational regulation of amino acid metabolism. The *dir1* mutant also had lower abundance of other defense related metabolites, e.g., antibiotics and secondary metabolites (Figure 5B). These results can help explain the susceptibility of the *dir1* mutant and the critical role of DIR1 in plant defense response. In contrast to the *dir1* mutant, the wild type plants increased the levels of these defense-related metabolites.

**FIGURE 6:**
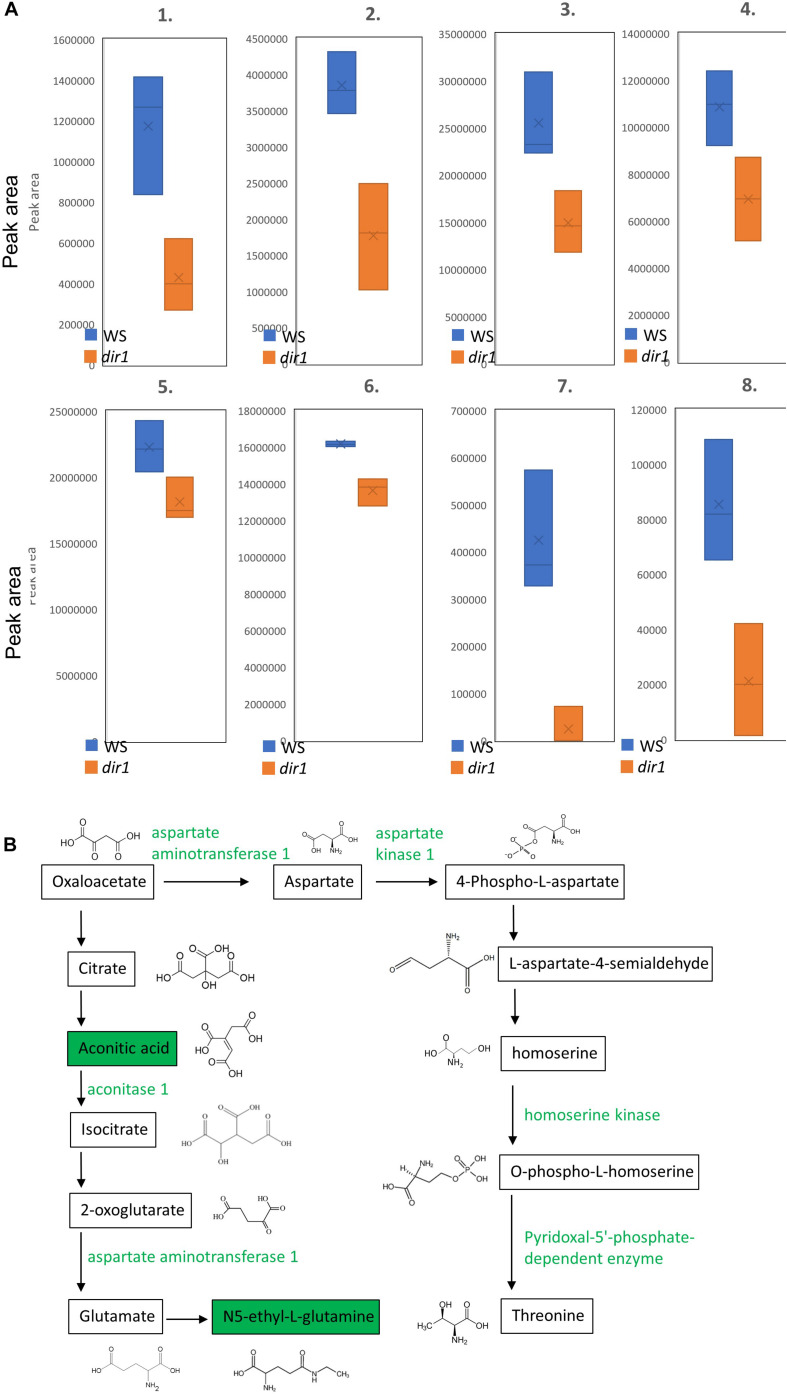
Amino acid biosynthesis pathways with differential metabolites and proteins in leaves of wild type and *dir1* mutant primed by *Pst* DC 3000 treatment. **(A)** Box plots showing lower abundance of six proteins and two metabolites in the glutamate and threonine biosynthetic pathways. 1. aspartate kinase 1; 2. homoserine kinase; 3. pyridoxal-5′-phosphate-dependent enzyme; 4. argininosuccinate synthase; 5. aconitase 1; 6. aspartate aminotransferase 1; 7. aconitic acid; 8. N5-ethyl-L-glutamine. **(B)** KEGG pathways of metabolites/proteins related to glutamate and threonine that are differentially abundant in wild type versus *dir1* primed leaves. Green color indicates decreased abundance.

Since amino acid metabolism was dramatically affected in the *dir1* mutant during SAR, here we focused on mapping proteins and metabolites onto the KEGG pathways for amino acid biosynthesis (Figure 6B). Six proteins and two metabolites were mapped to amino acid biosynthesis, and they were related to glutamate and glutamine, the top enriched pathway for the metabolite analysis (Figure 5A). All the six proteins and two metabolites were decreased in the primed *dir1* mutant when compared to primed wild type plants (Figure 6A). As amino acid biosynthesis is closely related to plant disease resistance (Zeier, 2013; Rojas et al., 2014; Erb and Kliebenstein, 2020), DIR1 may play a role in regulating amino acid during SAR priming. Amino acid metabolism is inhibited during the SAR response of the *dir1* mutant (Figure 5B). A previous metabolomic study revealed that the levels of several amino acids were significantly increased in *Arabidopsis* leaves inoculated with SAR-inducing *P.syringae*, including aromatic amino acids, branched-chain amino acids, Thr and Lys, whereas Asp was decreased (Zeier, 2013). Here we found a decrease in threonine biosynthesis in the *dir1* mutant (Figure 6B). Additionally, Kadotani et al. (2016) found that exogenous application of glutamate to rice leaves was sufficient to induce systemic resistance against rice blast. These results are consistent with our finding that compromised amino acid metabolism may contribute to the disease susceptibility of the *dir1* mutant. The potential connection between DIR1 and amino acid metabolism is a new discovery, which needs to be further characterized in future studies.

## Conclusion

Multi-omics has advanced our understanding of the complex molecular mechanisms underlying genetic diseases, host-pathogen interactions, and metabolic disorders important to human health and crop production. The 3-in-1 sample preparation method greatly facilitates application of proteomics, metabolomics and lipidomics technologies to tackling fundamental biological and systems biology questions. Here we demonstrated the utility and robustness of the improved method using *Arabidopsis* leaves from wild type and *dir1* mutant challenged with *Pseudomonas* pathogen that causes crop diseases. In total, we were able to profile 1849 proteins, 1967 metabolites and 424 lipids from single samples, and integrate them into pathways and networks. The high coverage of molecules has not been achieved before. In addition, integration of the data has generated interesting questions and testable hypotheses. For example, how DIR1 regulates amino acid metabolism is intriguing. Apparently, the extraction of proteins, metabolites and lipids simultaneously from the same sample (3-in-1) has the following advantages: (1) inexpensive and easy to perform as this method does not require any special reagents or kits; (2) reducing technical variations related to sample preparation of different molecules; (3) conservation of sample amount (e.g., in case of single-cell types and clinical biopsies); (4) enhancing multi-omics by high coverage, reproducibility and tight correlation between different biomolecules; (5) broadly applicable to any other cells or tissue types. Therefore, this newly improved method has great value to multi-omics and systems biology toward understanding cellular molecular networks (through hypothesis generation and hypothesis testing) important for biological functions, traits and phenotypes.

## Data Availability Statement

The datasets presented in this study can be found in online repositories. The names of the repository/repositories and accession number(s) can be found below: All protein MS raw data and search results have been deposited to the ProteomeXchange Consortium via the PRIDE partner repository with the data set identifier PXD023094. All metabolite and lipid MS raw data and search results have been deposited to the MetaboLights data repository with the data set identifier MTBLS2303.

## Author Contributions

SC and JC conceived the idea and designed and supervised the experiments. JK and LD did the sample preparation, acquired all the proteomics, metabolomics and lipidomics data, and analyzed the data. YL assisted with data analysis and interpretation. JK wrote the first draft of the manuscript. LD improved the writing. JK, YL, and SC revised the manuscript. SC finalized the manuscript for publication. All authors participated in data interpretation, manuscript preparation, and read and approved the final version of the manuscript.

## Conflict of Interest

The authors declare that the research was conducted in the absence of any commercial or financial relationships that could be construed as a potential conflict of interest.

## Supplementary Material

Supplementary Table 1 Proteins, metabolites, and lipids identified in SAR primed wild type (WS ecotype) and *dir1* knockout mutant leaves.

Supplementary Table 2 Comparison of the identified lipid classes and relative abundances with other published papers.
